# Clinical Outcomes Following Personalized Gut Microbiota-Targeted Therapy in Children with Atopic Dermatitis and Laboratory-Confirmed Dysbiosis: A Single-Arm Prospective Pilot Study

**DOI:** 10.3390/nu18152572

**Published:** 2026-08-06

**Authors:** Raluca-Gabriela Miulescu, Ioana Roşca, Ruxandra-Cristina Marin, Călin Muntean, Alexandru-Neculai Pavel, Smaranda Stoleru, Andreea Teodora Constantin, Elena Poenaru, Oana Andreea Parliteanu, Daniela Eugenia Popescu, Oana Andreia Coman

**Affiliations:** 1 Faculty of Medicine, University of Medicine and Pharmacy “Carol Davila”, 020021 Bucharest, Romania; miulescuraluca@yahoo.com (R.-G.M.); ioana.rosca@umfcd.ro (I.R.); smaranda.stoleru@umfcd.ro (S.S.); andreea.constantin@umfcd.ro (A.T.C.); elena.poenaru@umfcd.ro (E.P.); oana.coman@umfcd.ro (O.A.C.); 2 Dermatology Department, Saint Constantin Hospital, 500388 Brasov, Romania; 3 Clinical Hospital of Obstetrics and Gynecology “Prof. Dr. P.Sirbu”, 060251 Bucharest, Romania; 4 Department III Functional Science, Discipline of Medical Informatics and Biostatistics, “Victor Babes” University of Medicine and Pharmacy, 300041 Timisoara, Romania; 5 Pax Clinic, 020951 Bucharest, Romania; alexnpavel@yahoo.com; 6 Pediatrics Department, National Institute for Mother and Child Health “Alessandrescu-Rusescu”, 20382 Bucharest, Romania; 7 Faculty of Medicine, “Titu Maiorescu” University, 031593 Bucharest, Romania; oana_andreea@yahoo.com; 8 Department of Obstetrics-Gynecology and Neonatology, “Victor Babeș” University of Medicine and Pharmacy, 300041 Timisoara, Romania; popescu.daniela@umft.ro

**Keywords:** atopic dermatitis, gut dysbiosis, gut–skin axis, probiotics, prebiotics, children, SCORAD, POEM, pilot study, single-arm

## Abstract

Background/Objectives: Atopic dermatitis (AD) is a chronic inflammatory skin disease in which gut–skin axis dysregulation is increasingly implicated. We conducted a prospective pilot study to describe the clinical evolution of pediatric AD following individualized, stool-guided microbiota-targeted therapy and to explore whether baseline dysbiosis markers predict outcomes beyond baseline disease severity. Methods: Twenty-one children with AD and laboratory-confirmed gut dysbiosis were enrolled consecutively at a tertiary pediatric center in Romania in a single-arm prospective pilot study. Gut microbiota was assessed using targeted culture-based stool analysis, generating a Flora Index and a binary High Putrefaction Flora classification. Individualized treatment consisted of strain-specific probiotics, prebiotics, and antifungals when indicated. Disease severity was assessed using the Patient-Oriented Eczema Measure (POEM) and Scoring Atopic Dermatitis (SCORAD) at baseline, 30 days, and 90 days. Longitudinal changes were evaluated using repeated-measures ANOVA and hierarchical regression models. Results: High Putrefaction Flora was present in 71.4% of participants. Mean POEM decreased from 14.67 ± 5.22 at baseline to 7.10 ± 3.82 at 90 days, while mean SCORAD decreased from 41.66 ± 15.28 to 17.93 ± 11.68 (both *p* < 0.001). Approximately 76% of participants achieved a ≥50% reduction in POEM, and 71% achieved a ≥50% reduction in SCORAD. Individualized microbiota-targeted therapy was associated with substantial improvements in both clinical and patient-reported disease severity over the 90-day follow-up. Although children with High Putrefaction Flora exhibited higher disease severity scores in unadjusted analyses, baseline disease severity was the only significant predictor of 90-day outcomes. The Flora Index provided no independent explanatory value. Conclusions: In this exploratory single-arm pilot study, individualized microbiota-targeted therapy was associated with substantial and clinically meaningful improvement in AD severity over 90 days. Dysbiosis markers were associated with disease burden but did not independently predict outcomes, suggesting that they may function primarily as indicators of baseline severity rather than prognostic biomarkers. Because of the uncontrolled study design, causal inferences regarding treatment effects cannot be made, and these findings should be considered hypothesis-generating. Adequately powered randomized controlled trials are required to determine the efficacy of individualized microbiota-targeted interventions in pediatric atopic dermatitis.

## 1. Introduction

Atopic dermatitis (AD), or atopic eczema, is a chronic, relapsing inflammatory skin disease characterized by intense pruritus, xerosis, and recurrent eczematous lesions that markedly impair sleep, psychosocial functioning, and quality of life in affected children and their families [1,2]. AD typically begins in early childhood and frequently represents the first manifestation of the “atopic march”, preceding the development of food allergy, allergic rhinitis, and asthma [3]. Beyond its clinical manifestations, AD is increasingly recognized as a heterogeneous disorder encompassing multiple phenotypes and endotypes with distinct immunological, barrier-related, and microbial characteristics that may contribute to variability in disease progression and treatment response [4].

This heterogeneity has become particularly relevant in the era of precision medicine, where improved characterization of disease mechanisms may facilitate more individualized therapeutic strategies. Although many children experience partial remission during adolescence, the global prevalence and economic burden of pediatric AD continue to rise [5,6].

The pathogenesis of AD is multifactorial and involves interactions between epidermal barrier dysfunction, immune dysregulation, environmental exposures, and microbial ecosystems. Filaggrin deficiency promotes transepidermal water loss and allergen penetration, while a type-2–skewed immune response drives chronic cutaneous inflammation [7]. The skin microbiome is also altered, with *Staphylococcus aureus* overgrowth frequently associated with disease flares [8]. In addition, early-life factors influencing immune and microbial development, including antibiotic exposure, mode of delivery, and infant feeding practices, have been implicated in AD susceptibility [9].

Recent multi-omics studies integrating microbial, immunological, and metabolic data have further highlighted the biological heterogeneity of pediatric AD and support the concept of the disease as a systemic disorder involving dynamic interactions between barrier function, immune regulation, metabolism, and host–microbiome interactions [10,11].

The gut–skin axis has emerged as a biologically plausible contributor to AD pathogenesis [12]. Infants who subsequently develop AD or allergic sensitization often exhibit reduced gut microbial diversity, depletion of beneficial taxa such as *Bifidobacterium* and *Lactobacillus*, expansion of potentially pro-inflammatory microorganisms, and alterations in microbial pathways involved in short-chain fatty acid (SCFA) production [13,14].

Functional alterations in the intestinal microbiota, including disturbances in SCFA metabolism, microbial metabolites, epithelial barrier integrity, and immune-regulatory pathways, have also been associated with disease severity [15,16,17]. Although these observations suggest potential biological links between intestinal dysbiosis and cutaneous inflammation, the precise mechanisms remain incompletely understood.

Several observational studies have reported associations between gut microbial alterations and AD severity, suggesting that dysbiosis may represent both a marker and a potential contributor to disease burden [18]. Additional evidence indicates that alterations in microbial composition and function may influence host immune homeostasis, although these mechanisms have not yet been fully elucidated [19].

The therapeutic modulation of intestinal microbial communities has therefore emerged as a promising strategy for restoring host–microbiome homeostasis in inflammatory disorders [20]. Research further suggests that microbiome-associated functional changes may correlate more closely with disease activity than microbial composition alone, highlighting the potential clinical relevance of both taxonomic and functional aspects of dysbiosis [21]. These observations have stimulated growing interest in microbiota-targeted interventions for AD. Combinations of probiotic microorganisms and prebiotic substrates may exert synergistic effects on microbial ecosystem modulation and host immune homeostasis, providing a rationale for microbiota-directed therapeutic strategies [22]. However, most randomized trials and meta-analyses have evaluated standardized probiotic formulations, yielding modest and heterogeneous effects that appear to depend on strain selection, patient characteristics, and baseline disease severity [23,24]. Although several pediatric studies have reported improvements following supplementation with selected *Lactobacillus*- and *Bifidobacterium*-based formulations, results remain inconsistent across populations and intervention protocols [15].

While microbiome research has increasingly focused on identifying microbial signatures associated with disease activity, considerably less attention has been directed toward determining whether dysbiosis-related markers provide clinically useful prognostic information beyond established clinical indicators. Although numerous studies have demonstrated associations between microbial alterations and AD severity, relatively few have examined whether microbiome-derived measures independently predict subsequent clinical outcomes or treatment response [25,26].

Furthermore, individualized microbiota-directed interventions guided by documented microbiological results remain uncommon, and longitudinal studies integrating baseline dysbiosis assessment with repeated validated clinical outcome measures are limited [10].

Although high-throughput sequencing technologies have substantially advanced microbiome research, their cost, technical complexity, and limited availability continue to restrict routine clinical implementation in many healthcare settings. In this context, targeted culture-based stool profiling may provide a pragmatic and clinically accessible approach for informing individualized microbiota-directed interventions [11].

This knowledge gap is clinically important because the utility of microbiome assessment depends not only on demonstrating biological associations but also on establishing whether microbiological markers provide actionable information beyond conventional clinical evaluation.

The present study aimed to characterize the clinical course of children with atopic dermatitis (AD) and laboratory-confirmed gut dysbiosis and to evaluate whether baseline dysbiosis markers were associated with disease severity and provided prognostic information beyond baseline disease severity. Given the exploratory uncontrolled design, the findings should be considered hypothesis-generating and are intended to inform the design of future controlled studies evaluating personalized microbiota-directed interventions in pediatric AD. The gut–skin axis mechanisms outlined above, including microbial diversity, microbial metabolites, short-chain fatty acids, and immune-regulatory pathways, are presented solely as biological background and were not directly investigated in the present study, which relied exclusively on targeted culture-based stool analysis.

## 2. Materials and Methods

### 2.1. Study Design

This single-center, single-arm, prospective pre–post interventional exploratory pilot study was conducted in the Dermatology Department of Saint Constantin Hospital, Brașov, Romania, between September 2025 and February 2026. The study aimed to evaluate the clinical evolution of children with AD and laboratory-confirmed gut dysbiosis following individualized microbiota-targeted therapy. Given its exploratory pilot nature, no placebo, standard-care, or active comparator group was included.

Participants were recruited consecutively during routine clinical practice. All children meeting the eligibility criteria during the recruitment period whose parents or legal guardians provided informed consent were invited to participate. The study was specifically designed to generate preliminary estimates of treatment effect sizes and to explore potential associations between baseline dysbiosis markers and clinical outcomes, thereby informing the design of future adequately powered randomized controlled trials rather than establishing causal treatment effects.

Clinical assessments and stool microbiological investigations were performed as part of routine clinical evaluation, while participation in the study involved the prospective collection and analysis of these data for research purposes.

### 2.2. Participants

Children were eligible for inclusion if they were younger than 18 years of age, had a diagnosis of AD of any severity established by a dermatologist according to the Hanifin and Rajka diagnostic criteria [27], and presented with laboratory-confirmed gut dysbiosis identified through stool microbiological assessment performed during the same clinical episode. Because laboratory-confirmed dysbiosis was an eligibility criterion, all enrolled participants exhibited microbiological abnormalities at baseline.

Children were excluded if they had any form of eczema or inflammatory skin disease other than AD, another autoimmune dermatological disorder, an active systemic infection, or recent exposure to systemic antibiotics, antifungals, probiotics, or synbiotics within the preceding four weeks. Patients with incomplete clinical data or loss to follow-up before the first post-baseline assessment were also excluded.

**Figure 1:**
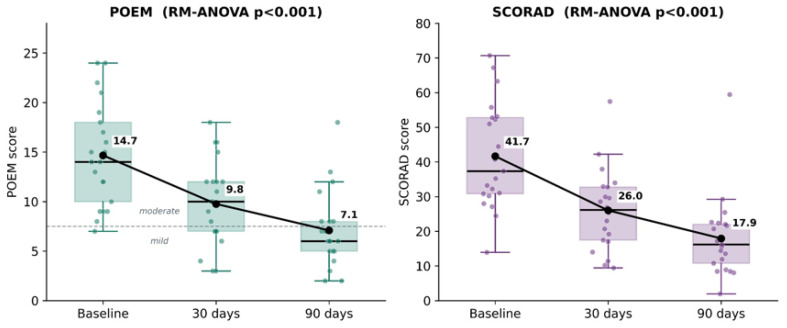
Evolution of POEM and SCORAD scores from baseline to 90 days. Boxplots show the median, interquartile range, and individual observations at baseline, 30 days, and 90 days. The black line connects cohort mean values. The dashed line in the POEM panel indicates the threshold for moderate disease severity.

A total of 21 children fulfilled all eligibility criteria and were included in the study. Participants were enrolled consecutively during the recruitment period, and all eligible patients whose parents or legal guardians provided informed consent were included in the analysis. The participant selection process, follow-up, and analysis population are summarized in the participant flow diagram (Figure 1). All enrolled participants completed the baseline, 30-day, and 90-day assessments, and no participant was lost to follow-up after enrolment.

No formal a priori sample-size calculation was performed because of the exploratory pilot nature of the study. The final sample comprised all eligible and consenting patients presenting during the recruitment period.

### 2.3. Stool Microbiological Assessment and Dysbiosis Classification

A single stool sample was collected from each participant at baseline, placed in a sterile collection container, stored at 2–8 °C, and transported to the reference laboratory within 24 h of collection. Gut microbial status was assessed using a targeted culture-based bacteriological and mycological stool examination supplemented by biochemical and physicochemical stool profiling. Molecular microbiome profiling techniques, including 16S rRNA gene sequencing and shotgun metagenomics, were not performed. Laboratory analyses were carried out according to the laboratory’s routine clinical diagnostic procedures. Laboratory personnel were not provided with the participants’ clinical severity scores (POEM or SCORAD), and the microbiological assessment was completed independently of the clinical outcome evaluation.

The laboratory analysis included semi-quantitative assessment of selected microbial groups commonly associated with intestinal eubiosis and dysbiosis. Evaluated proteolytic (putrefactive) bacteria included *Escherichia coli*, *Proteus* spp., *Klebsiella* spp., *Enterobacter* spp., *Hafnia alvei*, *Serratia* spp., *Providencia* spp., *Morganella morganii*, *Kluyvera* spp., *Citrobacter* spp., *Pseudomonas* spp., and *Clostridium* spp. Acidifying bacterial populations included *Bacteroides* spp., *Bifidobacterium* spp., *Lactobacillus* spp., and *Enterococcus* spp. Mycological assessment included *Candida albicans*, other *Candida* species, *Geotrichum* spp., and mould fungi.

Two laboratory-derived indicators of dysbiosis were used in the present analyses. The Flora Index was a composite ordinal score generated by the reference laboratory that integrated the degree of depletion of acidifying bacterial populations, the relative abundance of proteolytic microorganisms, stool pH, and the presence of fungal overgrowth or histamine-producing organisms. Higher values indicated a greater degree of dysbiosis. In addition, the laboratory reported the presence or absence of High Putrefaction Flora, a binary indicator reflecting marked predominance of proteolytic bacterial populations exceeding the laboratory’s internal reference threshold. Overall dysbiosis severity was also classified by the laboratory as mild, intermediate, or severe according to predefined internal criteria.

Because the Flora Index and the mild/intermediate/severe dysbiosis grading are proprietary composite measures generated by the reference laboratory, the exact numerical scoring algorithm and category thresholds were defined at laboratory discretion and could not be reproduced from an externally published standard, which limits reproducibility and independent verification. To maximize transparency despite this limitation, Supplementary Table S1 reports the individual microbiological findings underlying each patient’s laboratory assessment together with the corresponding Flora Index value and dysbiosis category assigned by the laboratory.

### 2.4. Clinical Evaluation

Disease severity and patient-reported disease burden were assessed at each study visit using the Patient-Oriented Eczema Measure (POEM) and the Scoring Atopic Dermatitis (SCORAD) index. POEM is a validated seven-item questionnaire that evaluates the frequency of eczema-related symptoms during the preceding week, including pruritus, sleep disturbance, bleeding, weeping, cracking, flaking, and skin dryness. Total scores range from 0 to 28, with higher scores indicating greater disease burden [28].

For children younger than 5 years of age, the questionnaire was completed by a parent or legal guardian, whereas older children completed the questionnaire with caregiver assistance when necessary.

Clinical disease severity was assessed using the SCORAD index, a validated composite measure incorporating the extent of skin involvement, lesion intensity, and subjective symptoms, including pruritus and sleep disturbance. Higher SCORAD scores indicate more severe disease activity [29].

Assessments were performed at baseline prior to initiation of individualized microbiota-targeted therapy, after approximately 30 days of treatment (25–35-day window), and after approximately 90 days of treatment (80–100-day window).

Additional baseline variables collected for descriptive and exploratory analyses included age, sex, gestational status (term or preterm birth), mode of delivery (vaginal delivery or cesarean section), conception by in vitro fertilization, and history of any breastfeeding (yes/no).

### 2.5. Intervention

Following baseline stool microbiological assessment, each child received an individualized microbiota-targeted regimen prescribed by the treating dermatologist or pediatrician according to the laboratory findings. The therapeutic approach aimed to replenish depleted acidifying microbial populations and, when indicated, address fungal overgrowth. Treatment selection was guided by the microbiological profile identified in each stool sample. No formal prespecified treatment algorithm was used; therapeutic decisions were based on the stool microbiological profile and routine clinical judgment. Participants continued their routine atopic dermatitis management throughout the study. No study-mandated modifications of conventional dermatological therapy were introduced, and decisions regarding emollients, topical corticosteroids, topical calcineurin inhibitors, rescue therapy, or other standard treatments remained entirely at the discretion of the treating physician. Changes in conventional therapy during follow-up were neither standardized nor systematically recorded. Microbiota-targeted interventions typically included one or more probiotic preparations containing *Lactobacillus* spp., *Bifidobacterium* spp., and *Enterococcus* spp. In selected cases, additional preparations containing *Bacteroides* spp., *Lactococcus* spp., or *Streptococcus thermophilus* were prescribed according to the stool microbiological results. Prebiotic supplementation with inulin or oligofructose was used when enhancement of beneficial microbial populations was considered appropriate, whereas oral fluconazole was prescribed for children with documented *Candida* overgrowth or elevated fungal burden.

Across the cohort, the prescribed probiotic regimens contained *Enterococcus* spp. (n = 19), *Lactobacillus* spp. (n = 13), *Bifidobacterium* spp. (n = 7), and *Bacteroides* spp. (n = 4), with *Streptococcus thermophilus* and *Lactococcus* spp. each prescribed in a single child; prebiotic inulin was added in two children and oral fluconazole in three children with documented fungal overgrowth. Exact probiotic strains, commercial formulations, colony-forming-unit doses, and treatment duration were not standardized or systematically recorded across this individualized regimen, representing a limitation for reproducibility.

Because the intervention was individualized, no single formulation or fixed therapeutic protocol was applied across the cohort. The microbiological outcomes and corresponding microbiota-targeted regimens for each participant are presented in Supplementary Table S1. Standard dermatological care, including emollients and topical anti-inflammatory therapies when clinically indicated, was continued throughout the study. Adherence to the prescribed microbiota-targeted regimen was assessed by caregiver report at follow-up visits. Assessment of treatment safety was not a predefined study endpoint, and adverse events were not prospectively collected using a structured assessment instrument. Likewise, follow-up stool microbiological assessments were not routinely performed during the study period.

### 2.6. Statistical Analysis

Continuous variables are presented as mean ± standard deviation (SD) or median (interquartile range, IQR), as appropriate, while categorical variables are presented as counts and percentages. Changes in POEM and SCORAD scores across the three study visits were evaluated using repeated-measures analysis of variance (ANOVA). Sphericity was assessed using Mauchly’s test, and the Greenhouse–Geisser correction was applied when the assumption of sphericity was violated. Effect sizes are reported as partial η^2^ for omnibus time effects and paired Cohen’s dz for baseline-to-90-day changes. Pairwise comparisons between time points were adjusted using the Bonferroni method.

Associations between baseline High Putrefaction Flora status and POEM or SCORAD scores were examined using the Mann–Whitney U test (exact method). Associations between the continuous Flora Index and disease severity or degree of improvement were evaluated using Spearman’s rank correlation coefficient.

To explore predictors of clinical outcomes at 90 days, hierarchical linear regression analyses were performed using three nested models. Model 1 included the corresponding baseline POEM or SCORAD score. Model 2 additionally included the Flora Index as the primary dysbiosis-related variable. Model 3 further incorporated age and breastfeeding status and was considered exploratory because of the limited sample size. Model performance was evaluated using R^2^, adjusted R^2^, and changes in explained variance (ΔR^2^). Standardized regression coefficients (β) are reported. The same modelling strategy was applied to the 30-day outcomes as a secondary analysis.

Additional exploratory and sensitivity analyses were conducted to evaluate the robustness of the findings, including responder analyses using multiple clinically relevant definitions, sensitivity analyses based on baseline severity and exclusion of a non-responder, bootstrap validation, ridge regression, and leave-one-out cross-validation. Individual patient trajectories were visualized using spaghetti plots to illustrate inter-individual variability in treatment response.

A two-sided *p*-value < 0.05 was considered statistically significant. All statistical analyses were performed using JASP version 0.18.3 (JASP Team, University of Amsterdam, Amsterdam, The Netherlands), with R version 4.3 serving as the computational backend. To enhance transparency and reproducibility, de-identified individual participant data underlying the analyses are provided in Supplementary Table S2, and additional study data may be made available by the corresponding authors upon reasonable request, subject to institutional ethics requirements and applicable data-sharing agreements. The within-subject change in POEM and SCORAD from baseline to Day 90 was regarded as the primary outcome; the 30-day comparisons, subgroup and correlation analyses, and regression Models 2 and 3 were considered secondary or exploratory and were not formally prespecified. All prespecified analyses were performed on the complete-case dataset. All 21 enrolled participants completed the baseline, 30-day, and 90-day assessments; therefore, there were no missing outcome data and no imputation procedures were required. The study was not prospectively registered, and its objectives were primarily those of a feasibility pilot—recruitment, retention, adherence, and safety—rather than a confirmatory efficacy trial.

### 2.7. Ethical Considerations

The study was conducted in accordance with the ethical principles of the Declaration of Helsinki and was approved by the Institutional Review Board (Ethics Committee) of Saint Constantin Hospital, Brașov, Romania (approval no. 36/27 August 2025). Written informed consent was obtained from the parents or legal guardians of all participants before enrollment.

Participation in the study was voluntary, and caregivers were informed that refusal to participate or withdrawal from the study at any stage would not affect the medical care received. All clinical and microbiological data were anonymized before analysis to ensure participant confidentiality and data protection. Safety outcomes were not predefined study endpoints and were not collected using a structured adverse-event reporting instrument. No treatment-related adverse events were spontaneously reported by caregivers during follow-up. However, the absence of systematic safety monitoring should be considered a limitation of the present pilot study.

## 3. Results

### 3.1. Sample Characteristics

Twenty-one children with atopic dermatitis (AD) and laboratory-confirmed gut dysbiosis were included in the study (Table 1). The mean age was 4.86 ± 3.75 years (median 3 years, IQR 2–6; range 1–15 years), and 47.6% (n = 10) were male. Cesarean delivery was reported in 18 children (85.7%), 9 (42.9%) had been born preterm, 1 (4.8%) had been conceived by in vitro fertilization, and 9 (42.9%) had a history of breastfeeding.

Regarding microbiological results, High Putrefaction Flora was identified in 15 children (71.4%), and the median Flora Index was 5 (IQR 4–6; range 2–9). Dysbiosis severity was classified as mild in 12 children (57.1%) and intermediate in 9 children (42.9%); no participant was classified as having severe dysbiosis.

At baseline, the mean POEM score was 14.67 ± 5.22 and the mean SCORAD score was 41.66 ± 15.28.

### 3.2. Evolution of POEM and SCORAD over Time

Descriptive statistics for POEM and SCORAD scores at baseline, 30 days, and 90 days are presented in Table 2, while the temporal evolution of both outcomes is illustrated in Figure 1. Mean POEM scores decreased from 14.67 ± 5.22 at baseline to 9.76 ± 4.29 at 30 days and 7.10 ± 3.82 at 90 days. Similarly, mean SCORAD scores decreased from 41.66 ± 15.28 at baseline to 26.02 ± 11.63 at 30 days and 17.93 ± 11.68 at 90 days.

Repeated-measures ANOVA demonstrated a significant effect of time for both outcome measures (Table 3). For POEM, the assumption of sphericity was met (Mauchly’s W = 0.740, *p* = 0.057), and a strong within-subject effect of time was observed (F(2,40) = 63.04, *p* < 0.001, partial η^2^ = 0.759). For SCORAD, sphericity was violated (Mauchly’s W = 0.556, *p* = 0.004), and the Greenhouse–Geisser correction was applied (ε = 0.693), yielding a similarly strong effect of time (F(1.39,27.71) = 59.56, *p* < 0.001, partial η^2^ = 0.749).

All pairwise comparisons remained statistically significant after Bonferroni adjustment (all *p* < 0.001; Table 3). The largest reductions were observed between baseline and 90 days, corresponding to mean decreases of 7.57 points for POEM (95% CI 5.45–9.69) and 23.73 points for SCORAD (95% CI 16.59–30.86). The paired effect sizes were very large (POEM dz = 2.03; SCORAD dz = 1.90), and the mean relative reductions from baseline to 90 days (approximately 52% for POEM and 57% for SCORAD) exceeded the established minimal clinically important differences for both instruments [30].

### 3.3. Association of Dysbiosis Markers with Disease Severity

**Figure 2:**
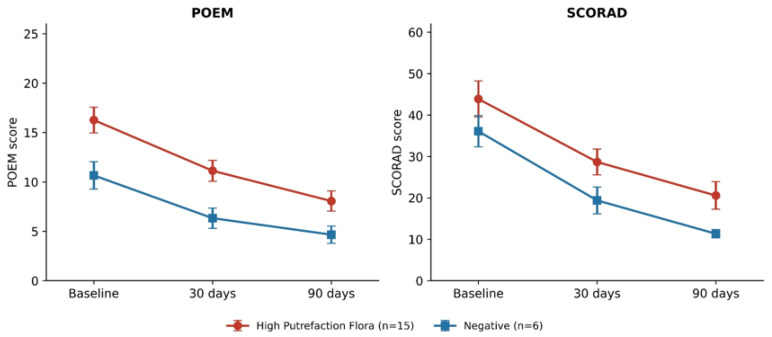
POEM and SCORAD trajectories according to baseline High Putrefaction Flora status. Values are presented as mean ± SEM at baseline, 30 days, and 90 days.

Associations between baseline High Putrefaction Flora status and disease severity are presented in Table 4 and illustrated in Figure 2. Children with High Putrefaction Flora had significantly higher POEM scores than those without this finding at baseline (median 15.0 [IQR 13.5–20.0] vs. 9.0 [9.0–11.25]; *p* = 0.023), 30 days (12.0 [8.5–13.5] vs. 7.0 [4.75–7.0]; *p* = 0.018), and 90 days (7.0 [6.0–9.5] vs. 4.5 [3.25–5.75]; *p* = 0.045) (Table 4).

For SCORAD, differences between groups were not statistically significant at baseline (44.45 [31.5–54.4] vs. 33.12 [30.4–39.4]; *p* = 0.302) or at 30 days (28.55 [23.1–33.4] vs. 18.12 [14.8–22.1]; *p* = 0.095), but reached significance at 90 days (20.70 [15.9–22.5] vs. 11.38 [9.4–13.2]; *p* = 0.037) (Table 4).

In contrast, the continuous Flora Index showed weak and non-significant correlations with baseline disease severity (POEM: ρ = −0.11, *p* = 0.64; SCORAD: ρ = −0.18, *p* = 0.45), as illustrated in Figure 2 Thus, whereas High Putrefaction Flora demonstrated significant unadjusted associations with disease severity, no significant associations were observed for the continuous Flora Index. These findings were further evaluated in multivariable analyses to determine whether dysbiosis-related measures contributed independently to clinical outcomes.

### 3.4. Baseline Predictors of 90-Day Outcomes

Results of the hierarchical linear regression analyses are presented in Table 5. For POEM, the baseline score alone explained 49.2% of the variance in the 90-day outcome (R^2^ = 0.492, *p* < 0.001; standardized β = 0.70). Addition of the Flora Index (Model 2) resulted in a negligible and non-significant increase in explained variance (R^2^ = 0.493; ΔR^2^ = 0.001; *p* = 0.843; Flora Index β = −0.03). Further adjustment for age and breastfeeding status (Model 3) produced only a marginal increase in model fit (R^2^ = 0.508; ΔR^2^ = 0.015; *p* = 0.788).

A similar pattern was observed for SCORAD. Baseline SCORAD explained 35.8% of the variance in the 90-day score (R^2^ = 0.358, *p* = 0.004; β = 0.60), whereas the Flora Index contributed minimally (Model 2: ΔR^2^ = 0.003; *p* = 0.761), and additional demographic covariates did not improve model performance (Model 3: ΔR^2^ = 0.007; *p* = 0.911). Secondary analyses using the 30-day outcomes yielded comparable results, with no significant contribution of the Flora Index in either model (ΔR^2^ ≤ 0.006, *p* ≥ 0.65).

**Figure 3:**
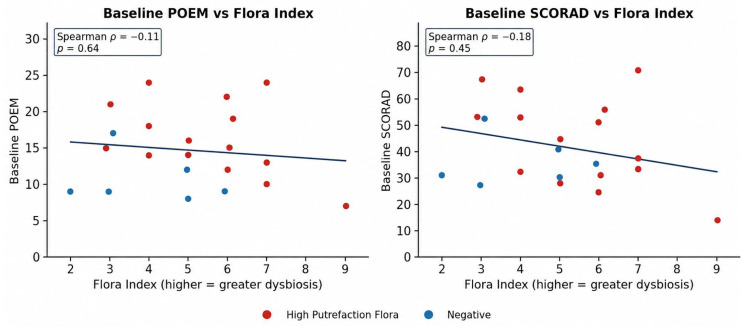
Relationship between the Flora Index and baseline POEM and SCORAD scores. Scatterplots with fitted regression lines illustrating the weak, non-significant associations between the Flora Index and baseline disease severity.

Across outcomes and time points, baseline disease severity was the only significant predictor of follow-up severity. Although High Putrefaction Flora showed significant unadjusted associations with disease severity (Section 3.3), these associations were not retained after accounting for baseline severity. Figure 3 further illustrates the absence of a meaningful relationship between the continuous Flora Index and baseline POEM or SCORAD scores.

Adjusted R^2^ values decreased as additional predictors were introduced into the models (Table 5). Bootstrap resampling, ridge regression, and leave-one-out cross-validation yielded findings consistent with the primary analyses, with no evidence that the Flora Index contributed substantial independent predictive information beyond baseline disease severity.

### 3.5. Additional Exploratory and Sensitivity Analyses

**Figure 4:**
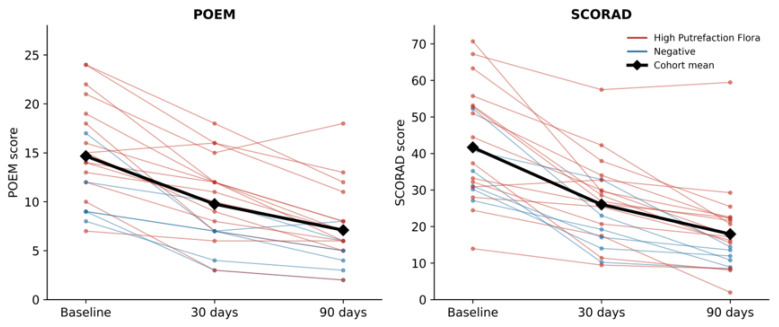
Individual trajectories of POEM and SCORAD scores during follow-up. Each line represents one participant, stratified according to baseline High Putrefaction Flora status. The thick black line represents the cohort mean.

Individual patient trajectories are presented in Figure 4. Although most children demonstrated progressive reductions in both POEM and SCORAD scores throughout follow-up, substantial inter-individual variability was observed. The majority of participants showed sustained improvement over time, whereas one child exhibited minimal overall change, highlighting the heterogeneity of treatment response within the cohort.

**Figure 5:**
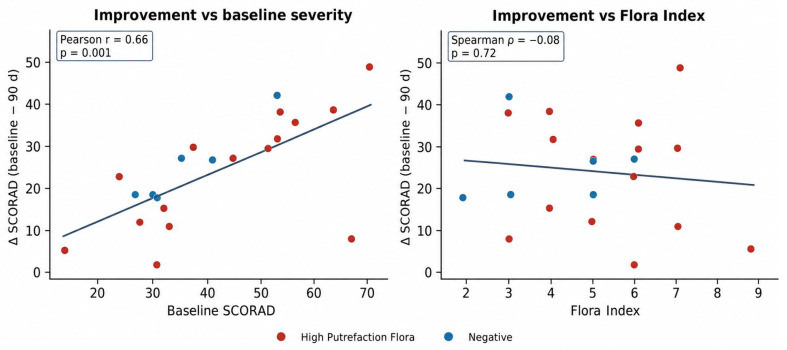
Associations between improvement in disease severity and baseline characteristics. Scatterplots showing the relationship between SCORAD improvement (baseline–90 days) and baseline disease severity (left panel) or Flora Index (right panel).

Children with High Putrefaction Flora showed a numerically greater mean improvement in POEM scores than those without this finding (ΔPOEM 8.20 ± 4.14 vs. 6.00 ± 1.79), although the difference did not reach statistical significance (*p* = 0.184; Cohen’s d = 0.60). No between-group difference was observed for SCORAD improvement (ΔSCORAD 23.32 ± 13.88 vs. 24.75 ± 9.20; *p* = 0.938; d = −0.11). Similarly, the Flora Index was not significantly correlated with the degree of improvement in either POEM (ρ = 0.03, *p* = 0.89) or SCORAD (ρ = −0.08, *p* = 0.72). As illustrated in Figure 5, greater baseline disease severity was associated with larger absolute improvements, whereas no clear relationship was observed between the Flora Index and treatment response.

Using the predefined ≥50% reduction criterion, 16 of 21 children (76.2%) achieved a POEM response and 15 of 21 (71.4%) achieved a SCORAD response by 90 days (Table 6). Responder rates increased to 85–91% when alternative clinically relevant definitions were applied, including achievement of the minimal clinically important difference (MCID) and the ≥30% SCORAD reduction threshold. Responder rates did not differ significantly according to High Putrefaction Flora status (POEM: 73% vs. 83%, Fisher’s exact *p* = 1.000; SCORAD: 60% vs. 100%, *p* = 0.123).

Sensitivity analyses demonstrated that exclusion of the single non-responder produced only minimal changes in the degree of improvement (ΔPOEM 7.57 → 7.80; ΔSCORAD 23.73 → 24.53), and all primary within-subject comparisons remained statistically significant (*p* < 0.001). Children with higher baseline disease severity (SCORAD > 40; n = 10) experienced larger absolute improvements than those with lower baseline severity, with mean reductions of 10.0 versus 5.4 points for POEM and 32.1 versus 16.1 points for SCORAD.

Given the limited sample size, the subgroup, correlation, and multivariable analyses should be considered exploratory and interpreted with caution. Emphasis should therefore be placed on the magnitude and precision of the observed effect sizes, together with their corresponding confidence intervals, rather than on statistical significance alone. Consequently, non-significant subgroup and predictor analyses should be regarded as inconclusive rather than evidence of no association and should primarily be considered hypothesis-generating. Interpretation should therefore focus primarily on the magnitude and precision of the observed effect sizes and their corresponding 95% confidence intervals rather than on statistical significance alone.

Additional statistical analyses supporting the robustness of the main findings, including effect sizes, responder analyses, sensitivity analyses, complementary regression diagnostics, and illustrative sample-size estimations for future randomized trials, are presented in Supplementary Note S1.

## 4. Discussion

In this single-arm prospective pilot study of children with atopic dermatitis (AD) and laboratory-confirmed gut dysbiosis, individualized microbiota-targeted management delivered alongside routine dermatological care was associated with substantial improvements in both clinician-assessed disease severity (SCORAD) and patient/caregiver-reported disease burden (POEM) over 90 days. Improvements were consistently observed across repeated-measures, responder, and sensitivity analyses, with most participants demonstrating progressive reductions in disease severity throughout follow-up. Approximately three-quarters of participants achieved a ≥50% reduction in POEM or SCORAD scores, while more than 85% reached the validated minimal clinically important difference thresholds. Nevertheless, individual trajectories showed considerable variability in treatment response, highlighting the heterogeneous clinical course of pediatric AD. Given the uncontrolled study design, these findings should be interpreted as hypothesis-generating and should not be considered evidence of treatment efficacy. An additional consideration when interpreting these findings is that all participants continued routine dermatological management throughout the study, while changes in concomitant therapies, including emollients, topical anti-inflammatory treatments, and rescue medications, were neither standardized nor systematically documented. Consequently, the respective contribution of conventional dermatological therapy and the individualized microbiota-targeted intervention to the observed clinical improvement cannot be distinguished within the present uncontrolled study design. The observed improvements should therefore be interpreted as reflecting the overall management strategy rather than the microbiota-targeted intervention alone.

A principal finding of this study was the difference between the unadjusted and adjusted analyses of the evaluated dysbiosis markers. Children with High Putrefaction Flora consistently exhibited higher POEM scores throughout follow-up, whereas associations with SCORAD were less consistent and reached statistical significance only at the final assessment. In contrast, the continuous Flora Index was not significantly associated with baseline disease severity, subsequent clinical improvement, or responder status. Furthermore, after adjustment for baseline disease severity, neither dysbiosis marker contributed independent prognostic information, while baseline disease severity remained the only significant predictor of 90-day outcomes. Together, these findings suggest the evaluated culture-based dysbiosis markers were associated with disease burden in unadjusted analyses but did not provide independent prognostic information in this exploratory cohort. Given the limited sample size, these findings should be interpreted as inconclusive rather than as evidence that the evaluated microbiological markers lack prognostic or biological relevance. This observation is consistent with recent evidence indicating that, although microbiome-related factors contribute to AD pathophysiology and are frequently associated with disease severity, their incremental prognostic value beyond established clinical characteristics is generally limited [31].

These findings are consistent with research indicating that associations between gut microbial alterations and AD severity do not necessarily translate into clinically useful prognostic biomarkers. Recent pediatric studies have reported significant relationships between microbial composition, microbial metabolic pathways, and disease severity, including alterations in short-chain fatty acid–producing taxa and other microbiome-related functions associated with higher SCORAD scores [32]. Likewise, coordinated disturbances across gut, skin, and oral microbial communities have been associated with disease severity, although considerable inter-individual variability has been observed [33]. Similar observations have been reported in studies demonstrating associations between microbial diversity, community composition, functional pathways, and inflammatory activity in children with AD [34]. Taken together, these data support a relationship between gut dysbiosis and disease burden, while suggesting that currently available microbiological markers may have more limited value for predicting future clinical outcomes.

Children with greater baseline disease severity also experienced larger absolute improvements in both POEM and SCORAD during follow-up. This finding is not unexpected because participants with more severe disease had greater scope for measurable improvement, and baseline severity consistently emerged as the strongest predictor of subsequent clinical outcomes. Importantly, in the absence of a control group, these observations should not be interpreted as evidence that children with more severe AD derive greater benefit from individualized microbiota-targeted management. Longitudinal cohort studies likewise suggest that microbiome-related factors contribute to disease susceptibility and clinical expression but provide only modest prognostic information when considered alongside established clinical and environmental determinants [35]. Accordingly, the present findings support the view that dysbiosis represents one component of a multifactorial disease process, whereas baseline clinical severity remains the strongest predictor of short-term outcomes in this cohort.

Although increasing evidence supports a biological link between the gut microbiota and atopic dermatitis, the mechanisms underlying the gut–skin axis were not directly investigated in the present study, which relied exclusively on targeted culture-based stool analysis. Recent sequencing-based studies have reported associations between gut microbial composition, microbial metabolic pathways, and disease severity, including alterations involving butyrate-producing bacteria and sphingolipid-related pathways [32]. Coordinated disturbances across gut, skin, and oral microbial communities, together with alterations in inflammatory biomarkers, have also been described in pediatric AD [33,36]. These observations provide biological context for microbiota-targeted approaches but should not be interpreted as mechanistic evidence supporting the findings of the present study.

The extent of clinical improvement observed in the present cohort is broadly consistent with previous studies evaluating microbiota-targeted interventions in pediatric AD. Randomized and controlled trials have reported reductions in disease severity following supplementation with selected *Lactobacillus*- and *Bifidobacterium*-based probiotic formulations, in some cases accompanied by improvements in quality of life and changes in gut microbial composition [37,38,39]. Additional pediatric studies have reported favorable clinical and immunological effects of microbiota-directed interventions in allergic diseases, further supporting the potential biological relevance of microbiome modulation during early life [40]. However, because the probiotic formulations, patient populations, and study designs differed substantially across studies, direct comparisons should be interpreted cautiously.

In addition to strain-specific probiotics, a small number of participants received inulin as part of the individualized intervention. Inulin-type fructans promote the growth and metabolic activity of beneficial intestinal microorganisms and have demonstrated favorable effects in several pediatric gastrointestinal disorders [41]. Although these findings cannot be directly extrapolated to atopic dermatitis, they provide additional biological rationale for including prebiotic supplementation within individualized microbiota-targeted management strategies.

Nevertheless, comparisons with previous intervention studies remain limited because the present investigation lacked a control group and employed individualized rather than standardized microbiota-targeted regimens. Furthermore, combinations of microbial strains were prescribed according to each participant’s microbiological profile, whereas most published randomized trials have evaluated fixed probiotic formulations. Although multi-strain preparations may provide complementary biological effects and have attracted increasing interest across several biomedical fields [42], the present study cannot determine the relative contribution of any individual probiotic strain, prebiotic component, or antifungal therapy to the observed clinical outcomes.

The limited prognostic value of the Flora Index may partly reflect the characteristics of the microbiological assessment itself. The Flora Index was derived from a targeted culture-based assay and therefore provides only a broad estimate of intestinal microbial imbalance. Unlike sequencing-based approaches, culture-based methods do not characterize microbial diversity, community structure, functional gene content, metabolite production, or host–microbiome interactions [43,44]. Accordingly, the absence of an independent association between the Flora Index and clinical outcomes should not be interpreted as evidence against the biological relevance of the gut–skin axis, but rather as an indication that the evaluated culture-based markers may not adequately capture the functional aspects of dysbiosis most relevant to disease activity and prognosis [45,46,47].

The cohort was characterized by a high prevalence of cesarean delivery and prematurity and a relatively low prevalence of breastfeeding. Although the present study was not designed to evaluate the influence of these factors on disease severity or treatment response, these characteristics are noteworthy because early-life microbial exposures have been associated with immune maturation and susceptibility to allergic diseases [48,49,50]. Likewise, associations between gut microbial alterations and markers of allergic inflammation have been described in pediatric populations [51,52]. While no causal conclusions can be drawn from the present findings, these observations are consistent with the multifactorial nature of atopic dermatitis, in which host, environmental, immunological, and microbial factors interact throughout disease development [53].

Several limitations should be considered when interpreting the findings of this study. The uncontrolled single-arm design precludes causal inference. Consequently, the observed clinical improvements cannot be attributed specifically to the microbiota-targeted intervention and may partly reflect the natural fluctuating course of AD, regression to the mean, seasonal variation, increased clinical attention, improved adherence to standard care, concomitant standard dermatological therapy, caregiver expectation and placebo-related effects, unmeasured dietary modifications occurring during follow-up, or other unmeasured co-interventions. Regression to the mean is particularly relevant because participants were enrolled during periods of active disease and baseline severity emerged as the strongest predictor of subsequent outcomes. The small sample size and single-center recruitment further limit generalizability and reduced statistical power for subgroup analyses, between-group comparisons, and identification of independent predictors of treatment response. Accordingly, non-significant findings, particularly those related to dysbiosis markers and responder subgroup analyses, should be interpreted as inconclusive rather than evidence of no association. Thus, the multivariable regression analyses should be interpreted as exploratory, hypothesis-generating analyses rather than as definitive predictive models. Because enrollment required laboratory-confirmed gut dysbiosis, the cohort represents a highly selected subgroup of children with atopic dermatitis; consequently, the findings cannot be generalized to the broader pediatric AD population, particularly children without laboratory-confirmed dysbiosis.

Additional limitations relate to microbiota assessment and intervention characterization. Gut microbial evaluation was based on a targeted culture-based assay rather than sequencing-based methods and therefore did not allow assessment of microbial diversity, community structure, functional capacity, or metabolite production. The culture-based dysbiosis assay used in this study has not been formally validated or standardized in pediatric populations. Consequently, its diagnostic performance, reproducibility, and external comparability remain uncertain, and the microbiological outcomes should therefore be interpreted with caution. In addition, oral fluconazole was administered to three children with documented fungal overgrowth according to routine clinical practice rather than a standardized protocol. Because antifungal therapy may influence both clinical symptoms and the intestinal microbial ecosystem, its use represents an additional source of treatment heterogeneity and potential confounding. Furthermore, follow-up stool analyses were not routinely performed, preventing evaluation of microbiological changes over time, while the absence of metabolomic, immunological, inflammatory, and intestinal permeability biomarkers limited mechanistic interpretation. Consequently, it was not possible to determine whether the individualized microbiota-targeted intervention resulted in correction of intestinal dysbiosis, altered the baseline microbiological profile, or whether any microbiota changes, if present, mediated the observed clinical improvements. Therefore, the observed clinical improvement should not be interpreted as evidence that intestinal dysbiosis was corrected. The intervention was individualized according to the baseline stool findings; however, detailed information regarding probiotic strains, commercial formulations, colony-forming-unit doses, and treatment duration was not systematically recorded, further limiting reproducibility. Because the intervention combined multiple, individually varying components delivered without a fixed protocol, the observed improvements cannot be attributed to any specific probiotic strain, prebiotic, or antifungal agent, and the heterogeneous treatment strategy substantially limits reproducibility. Concomitant standard dermatological care, including emollients, topical corticosteroids, topical calcineurin inhibitors, and any treatment modifications introduced during routine clinical care, was not standardized by the study protocol and was neither prospectively standardized nor systematically documented. Consequently, these therapies may independently account for part or all of the observed clinical improvement and represent a central source of potential confounding.

Assessment of treatment safety was not a predefined study endpoint, and adverse events were not prospectively collected using a structured assessment instrument. Although no treatment-related adverse events were spontaneously reported during follow-up, the absence of systematic safety monitoring precludes definitive conclusions regarding the safety or tolerability of the individualized microbiota-targeted intervention. In addition, intervention fidelity, participant acceptability, and predefined progression criteria were not formally evaluated, as these were not predefined objectives of this exploratory feasibility pilot. Their assessment should be incorporated into future randomized controlled trials.

Additional potentially relevant confounders, including baseline dietary intake and nutritional exposures (fibre intake, fermented foods, ultra-processed foods, food exclusions, and allergy-related dietary avoidance), prior antibiotic, probiotic, and dietary supplement use, environmental factors, socioeconomic characteristics, and dietary changes during follow-up, were not systematically assessed. Consequently, it cannot be determined whether the observed clinical improvements were related to the individualized microbiota-targeted intervention itself, concurrent dietary modifications, changes in overall diet quality, or other behavioral factors. The absence of structured dietary assessment represents a particular limitation given the nutritional focus of the present study. Finally, the 90-day follow-up period does not permit conclusions regarding long-term disease control, durability of response, microbiota stability, relapse prevention, or progression of the atopic march. Future randomized controlled studies should incorporate standardized dietary assessment together with standardized microbiota-targeted regimens, predefined probiotic strains, dosages, treatment duration, adherence monitoring, and repeated microbiological assessments to improve reproducibility and better distinguish the therapeutic contributions of nutritional and microbiota-directed interventions.

Despite these limitations, the present study has several strengths. Its prospective design enabled standardized longitudinal assessment using two validated and complementary outcome measures that captured both clinician-assessed disease severity and patient/caregiver-reported disease burden. All participants underwent predefined clinical assessments over the 90-day follow-up, allowing characterization of individual response trajectories. Methodological transparency was further enhanced through the presentation of individual participant data, multiple clinically relevant responder definitions, complementary sensitivity analyses, and exploratory regression models that distinguished unadjusted associations from independent predictors of outcome.

From a clinical perspective, these findings suggest that individualized microbiota-targeted management may represent a feasible adjunct within a broader multidisciplinary approach to pediatric AD. However, baseline disease severity consistently provided substantially greater prognostic information than the evaluated culture-based dysbiosis markers, indicating that careful clinical assessment remains the most informative predictor of short-term outcomes in this setting. Consequently, evidence-based skin-barrier restoration and anti-inflammatory therapy should remain the cornerstone of management, whereas microbiota-directed interventions should currently be regarded as investigational adjunctive strategies requiring confirmation in adequately powered randomized controlled trials.

## 5. Conclusions

In this single-arm prospective pilot study of children with atopic dermatitis and laboratory-confirmed gut dysbiosis, individualized microbiota-targeted therapy delivered alongside routine dermatological care was associated with clinically meaningful improvements in disease severity and patient-reported outcomes over 90 days. High Putrefaction Flora was associated with greater disease severity in unadjusted analyses, whereas the Flora Index did not independently predict outcomes after adjustment for baseline severity, suggesting that the evaluated dysbiosis markers primarily reflected disease burden.

Because of the uncontrolled study design, the observed improvements cannot be attributed specifically to the individualized microbiota-targeted intervention and should be regarded as hypothesis-generating rather than evidence of treatment efficacy. Accordingly, these findings should be considered hypothesis-generating rather than evidence of treatment efficacy. Nevertheless, this exploratory pilot study provides preliminary effect-size estimates that may inform sample-size calculations and the design of future adequately powered randomized controlled trials evaluating individualized microbiota-targeted interventions in pediatric atopic dermatitis.

## Figures and Tables

**Table 1:** Baseline demographic, microbiological, and clinical characteristics of the study cohort (N = 21).

Characteristic	n (%) or Mean ± SD/Median (IQR)
Sex	
Male	10 (47.6%)
Female	11 (52.4%)
Age (years)	
Mean ± SD	4.86 ± 3.75
Median (IQR; range)	3 (2–6; 1–15)
Perinatal and Early-Life Characteristics	
Cesarean delivery	18 (85.7%)
Preterm birth	9 (42.9%)
In vitro fertilization	1 (4.8%)
Any breastfeeding	9 (42.9%)
Microbiological Characteristics	
High Putrefaction Flora	15 (71.4%)
Flora Index, median (IQR; range)	5 (4–6; 2–9)
Dysbiosis severity—Mild	12 (57.1%)
Dysbiosis severity—Intermediate	9 (42.9%)
Dysbiosis severity—Severe	0 (0.0%)
Baseline Disease Severity	
Baseline POEM score, mean ± SD	14.67 ± 5.22
Baseline SCORAD score, mean ± SD	41.66 ± 15.28

IQR, interquartile range; POEM, Patient-Oriented Eczema Measure; SCORAD, Scoring Atopic Dermatitis.

**Table 2:** Descriptive statistics of POEM and SCORAD scores at baseline, 30 days, and 90 days (N = 21).

Outcome	Time Point	Minimum	Maximum	Mean ± SD	Median
POEM	Baseline	7	24	14.67 ± 5.22	14
POEM	30 days	3	18	9.76 ± 4.29	10
POEM	90 days	2	18	7.10 ± 3.82	6
SCORAD	Baseline	13.95	70.70	41.66 ± 15.28	37.35
SCORAD	30 days	9.45	57.45	26.02 ± 11.63	26.20
SCORAD	90 days	1.95	59.45	17.93 ± 11.68	16.20

POEM, Patient-Oriented Eczema Measure; SCORAD, Scoring Atopic Dermatitis.

**Table 3:** Repeated-measures ANOVA results and Bonferroni-adjusted pairwise comparisons for POEM and SCORAD scores.

Outcome/Contrast	Statistic	95% CI	*p*-Value
Omnibus time effect			
POEM	F(2,40) = 63.04; partial η^2^ = 0.759; Mauchly’s W = 0.740 (*p* = 0.057)	—	<0.001
SCORAD	F(1.39,27.71) = 59.56; partial η^2^ = 0.749; Mauchly’s W = 0.556 (*p* = 0.004); Greenhouse–Geisser ε = 0.693	—	
Pairwise mean differences			
POEM: Baseline–30 days	4.90	3.07–6.74	<0.001
POEM: Baseline–90 days	7.57	5.45–9.69	
POEM: 30 days–90 days	2.67	1.36–3.97	
SCORAD: Baseline–30 days	15.64	9.61–21.68	
SCORAD: Baseline–90 days	23.73	16.59–30.86	
SCORAD: 30 days–90 days	8.08	4.51–11.66	

CI, confidence interval.

**Table 4:** POEM and SCORAD scores stratified by baseline High Putrefaction Flora status (median [IQR]; exact Mann–Whitney U test).

Outcome	High Putrefaction Flora (n = 15)	Negative (n = 6)	*p*-Value
POEM—Baseline	15 [13.5–20.0]	9 [9.0–11.25]	0.023
POEM—30 days	12 [8.5–13.5]	7 [4.75–7.0]	0.018
POEM—90 days	7 [6.0–9.5]	4.5 [3.25–5.75]	0.045
SCORAD—Baseline	44.45 [31.5–54.4]	33.12 [30.4–39.4]	0.302
SCORAD—30 days	28.55 [23.1–33.4]	18.12 [14.8–22.1]	0.095
SCORAD—90 days	20.70 [15.9–22.5]	11.38 [9.4–13.2]	0.037

**Table 5:** Hierarchical linear regression models predicting 90-day POEM and SCORAD scores.

Model	Obs:Pred	R^2^	Adj. R^2^	ΔR^2^	*p*-Value (ΔR^2^)	Key Std. β (*p*-Value)
POEM at 90 days (primary: M1, M2)						
M1: Baseline POEM only	21:1	0.492	0.465	—	<0.001	Baseline 0.70 (<0.001)
M2: + Flora Index	10.5:1	0.493	0.437	0.001	0.843	Flora −0.03 (0.843)
M3: + Age + Breastfeeding (exploratory)	5.2:1	0.508	0.385	0.015	0.788	all *p* > 0.5
SCORAD at 90 days (primary: M1, M2)						
M1: Baseline SCORAD only	21:1	0.358	0.324	—	0.004	Baseline 0.60 (0.004)
M2: + Flora Index	10.5:1	0.361	0.290	0.003	0.761	Flora −0.06 (0.761)
M3: + Age + Breastfeeding (exploratory)	5.2:1	0.368	0.210	0.007	0.911	all *p* > 0.6

Adj. R^2^, adjusted coefficient of determination; ΔR^2^, change in explained variance; β, standardized regression coefficient. Model 3 should be interpreted as exploratory because of the limited sample size.

**Table 6:** Responder rates according to alternative clinical response definitions at 90 days (N = 21).

Definition	POEM	SCORAD
≥50% reduction (primary)	16/21 (76.2%)	15/21 (71.4%)
≥30% reduction	—	19/21 (90.5%)
MCID (POEM ≥ 3.4/SCORAD ≥ 8.7 points)	18/21 (85.7%)	18/21 (85.7%)
Final score in mild/clear range (POEM ≤ 7/SCORAD < 20)	14/21 (66.7%)	13/21 (61.9%)
≥70% reduction (stringent)	3/21 (14.3%)	—

## Data Availability

The de-identified dataset supporting the conclusions of this article is available from the first authors upon reasonable request.

## Supplementary Materials

The following supporting information can be downloaded at https://www.mdpi.com/article/10.3390/nu18152572/s1: Supplementary Table S1: Individualized microbiota-targeted therapy regimens and baseline microbiological findings (N = 21); Supplementary Table S2: Complete Individual Patient-Level Data (N = 21); Supplementary Note S1: Supplementary statistical analyses supporting the main findings, including effect sizes, responder analyses, repeated-measures analyses, exploratory subgroup analyses, sensitivity analyses, complementary regression diagnostics, and illustrative sample-size estimations for future randomized controlled trials.

## Abbreviations

The following abbreviations are used in this manuscript:

AD	Atopic dermatitis
ANCOVA	Analysis of covariance
BF	Breastfed
CFU	Colony-forming unit
CI	Confidence interval
C/S	Cesarean section
EASI	Eczema Area and Severity Index
FI	Flora Index
HPF	High Putrefaction Flora
IQR	Interquartile range
MCID	Minimal clinically important difference
POEM	Patient-Oriented Eczema Measure
Pre	Preterm
RCT	Randomized controlled trial
RespP	POEM responder
RespS	SCORAD responder
SCFA	Short-chain fatty acid
SCORAD	Scoring Atopic Dermatitis
Th2	Type 2 helper T-cell
ΔP	Change in POEM score
ΔS	Change in SCORAD score
P0	Baseline POEM score
P30	POEM score at 30 days
P90	POEM score at 90 days
S0	Baseline SCORAD score
S30	SCORAD score at 30 days
S90	SCORAD score at 90 days

## References

[1] Goksör E., Loid P., Alm B., Åberg N., Wennergren G. (2016). The allergic march comprises the coexistence of related patterns of allergic disease not just the progressive development of one disease. Acta Paediatr..

[2] Tian J., Zhang D., Yang Y., Huang Y., Wang L., Yao X., Lu Q. (2023). Global epidemiology of atopic dermatitis: A comprehensive systematic analysis and modelling study. Br. J. Dermatol..

[3] Li B., Fuxench Z.C. (2024). Atopic Dermatitis: Disease Background and Risk Factors. Adv. Exp. Med. Biol..

[4] Lupu V.V., Butnariu L.I., Fotea S., Morariu I.D., Badescu M.C., Starcea I.M., Salaru D.L., Popp A., Dragan F., Lupu A. (2023). The Disease with a Thousand Faces and the Human Microbiome—A Physiopathogenic Intercorrelation in Pediatric Practice. Nutrients.

[5] Adamson A.S. (2024). The Economic Impact of Atopic Dermatitis. Adv. Exp. Med. Biol..

[6] Manjelievskaia J., Boytsov N., Brouillette M.A., Onyekwere U., Pierce E., Goldblum O., Bonafede M. (2021). The direct and indirect costs of adult atopic dermatitis. J. Manag. Care Spec. Pharm..

[7] Irvine A.D., McLean W.H., Leung D.Y. (2011). Filaggrin mutations associated with skin and allergic diseases. N. Engl. J. Med..

[8] Geoghegan J.A., Irvine A.D., Foster T.J. (2018). Staphylococcus aureus and Atopic Dermatitis: A Complex and Evolving Relationship. Trends Microbiol..

[9] Yamamoto-Hanada K., Yang L., Narita M., Saito H., Ohya Y. (2017). Influence of antibiotic use in early childhood on asthma and allergic diseases at age 5. Ann. Allergy Asthma Immunol..

[10] Nekrasova A.I., Kalashnikova I.G., Bobrova M.M., Korobeinikova A.V., Bakoev S.Y., Ashniev G.A., Petryaikina E.S., Nekrasov A.S., Zagainova A.V., Lukashina M.V. (2024). Characteristics of the Gut Microbiota in Regard to Atopic Dermatitis and Food Allergies of Children. Biomedicines.

[11] Ilic I., Stojkovic A., Velickovic V., Zivanovic Macuzic I., Ilic M. (2023). Atopic Dermatitis in Children Under 5: Prevalence Trends in Central, Eastern, and Western Europe. Children.

[12] Simonyté Sjödin K., Hammarström M.L., Rydén P., Sjödin A., Hernell O., Engstrand L., West C.E. (2019). Temporal and long-term gut microbiota variation in allergic disease: A prospective study from infancy to school age. Allergy.

[13] Abrahamsson T.R., Jakobsson H.E., Andersson A.F., Björkstén B., Engstrand L., Jenmalm M.C. (2012). Low diversity of the gut microbiota in infants with atopic eczema. J. Allergy Clin. Immunol..

[14] Cait A., Cardenas E., Dimitriu P.A., Amenyogbe N., Dai D., Cait J., Sbihi H., Stiemsma L., Subbarao P., Mandhane P.J. (2019). Reduced genetic potential for butyrate fermentation in the gut microbiome of infants who develop allergic sensitization. J. Allergy Clin. Immunol..

[15] Pantazi A.C., Mihai C.M., Balasa A.L., Chisnoiu T., Lupu A., Frecus C.E., Mihai L., Ungureanu A., Kassim M.A.K., Andrusca A. (2023). Relationship between Gut Microbiota and Allergies in Children: A Literature Review. Nutrients.

[16] Roduit C., Frei R., Ferstl R., Loeliger S., Westermann P., Rhyner C., Schiavi E., Barcik W., Rodriguez-Perez N., Wawrzyniak M. (2019). High levels of butyrate and propionate in early life are associated with protection against atopy. Allergy.

[17] Akdis C.A. (2021). Does the epithelial barrier hypothesis explain the increase in allergy, autoimmunity and other chronic conditions?. Nat. Rev. Immunol..

[18] Ashkanani A., Ashkanani G., Yousef M., Rob M., Al-Marri M., Naseem N., Laws S., Chaari A. (2025). Microbiome and Skin Health: A Systematic Review of Nutraceutical Interventions, Disease Severity, Inflammation, and Gut Microbiota. Microorganisms.

[19] Kim J.E., Kim H.S. (2019). Microbiome of the Skin and Gut in Atopic Dermatitis (AD): Understanding the Pathophysiology and Finding Novel Management Strategies. J. Clin. Med..

[20] Sharma B., Agriantonis G., Twelker K., Ebelle D., Kiernan S., Siddiqui M., Soni A., Cheerasarn S., Simon W., Jiang W. (2025). Gut Microbiota Serves as a Crucial Independent Biomarker in Inflammatory Bowel Disease (IBD). Int. J. Mol. Sci..

[21] Blicharz L., Bukowska-Ośko I., Perlejewski K., Navarro-López V., Czuwara J., Waśkiel-Burnat A., Samochocki Z., Olszewska M., Rudnicka L., Radkowski M. (2026). Gut Microbiota Influence Host Metabolism and Immune Responses in Atopic Dermatitis: A Next-Generation Sequencing-Based Functional Profiling Study. Clin. Cosmet. Investig. Dermatol..

[22] Lee S., Choi S.-P., Choi H.-J., Jeong H., Park Y.-S. (2024). A comprehensive review of synbiotics: An emerging paradigm in health promotion and disease management. World J. Microbiol. Biotechnol..

[23] Xue X., Yang X., Shi X., Deng Z. (2023). Efficacy of probiotics in pediatric atopic dermatitis: A systematic review and meta-analysis. Clin. Transl. Allergy.

[24] Amalia N., Orchard D., Francis K.L., King E. (2020). Systematic review and meta-analysis on the use of probiotic supplementation in pregnant mother, breastfeeding mother and infant for the prevention of atopic dermatitis in children. Australas. J. Dermatol..

[25] Paller A.S., Kong H.H., Seed P., Naik S., Scharschmidt T.C., Gallo R.L., Luger T., Irvine A.D. (2019). The microbiome in patients with atopic dermatitis. J. Allergy Clin. Immunol..

[26] Bjerre R.D., Bandier J., Skov L., Engstrand L., Johansen J.D. (2017). The role of the skin microbiome in atopic dermatitis: A systematic review. Br. J. Dermatol..

[27] Hanifin J.M., Rajka G. (1980). Diagnostic features of atopic dermatitis. Acta Derm. Venereol. Suppl..

[28] Charman C.R., Venn A.J., Williams H.C. (2004). The patient-oriented eczema measure: Development and initial validation of a new tool for measuring atopic eczema severity from the patients’ perspective. Arch. Dermatol..

[29] European Task Force on Atopic Dermatitis (1993). Severity scoring of atopic dermatitis: The SCORAD index. Consensus Report of the European Task Force on Atopic Dermatitis. Dermatology.

[30] Schram M.E., Spuls P.I., Leeflang M.M., Lindeboom R., Bos J.D., Schmitt J. (2012). EASI, (objective) SCORAD and POEM for atopic eczema: Responsiveness and minimal clinically important difference. Allergy.

[31] Hilger E., Chan K., Yip A., Wang X.M., Broderick C., Arents B., van Bart K., Eyerich K., Rastrick J., Weidinger S. (2026). Biomarkers for therapeutic response and adverse outcomes in atopic dermatitis: A systematic review. J. Eur. Acad. Dermatol. Venereol..

[32] Liu X., Cai M., Chen M., Chen J., Zhu T., Wu S., Jia J. (2024). Alterations in gut microbiome associated with severity of atopic dermatitis in infants. Australas. J. Dermatol..

[33] Zhang X., Huang X., Zheng P., Liu E., Bai S., Chen S., Pang Y., Xiao X., Yang H., Guo J. (2024). Changes in oral, skin, and gut microbiota in children with atopic dermatitis: A case-control study. Front. Microbiol..

[34] Pavel F.M., Bungau S.G., Tit D.M., Ghitea T.C., Marin R.C., Radu A.F., Moleriu R.D., Ilias T., Bustea C., Vesa C.M. (2023). Clinical Implications of Dietary Probiotic Supplement (Associated with L-Glutamine and Biotin) in Ulcerative Colitis Patients’ Body Composition and Quality of Life. Nutrients.

[35] Hoskinson C., Medeleanu M.V., Reyna M.E., Dai D.L.Y., Chowdhury B., Moraes T.J., Mandhane P.J., Simons E., Kozyrskyj A.L., Azad M.B. (2024). Antibiotics taken within the first year of life are linked to infant gut microbiome disruption and elevated atopic dermatitis risk. J. Allergy Clin. Immunol..

[36] Kalashnikova I.G., Nekrasova A.I., Korobeynikova A.V., Bobrova M.M., Ashniev G.A., Bakoev S.Y., Zagainova A.V., Lukashina M.V., Tolkacheva L.R., Petryaikina E.S. (2024). The Association between Gut Microbiota and Serum Biomarkers in Children with Atopic Dermatitis. Biomedicines.

[37] de Andrade P.D.S.M.A., Maria E Silva J., Carregaro V., Sacramento L.A., Roberti L.R., Aragon D.C., Carmona F., Roxo-Junior P. (2021). Efficacy of Probiotics in Children and Adolescents with Atopic Dermatitis: A Randomized, Double-Blind, Placebo-Controlled Study. Front. Nutr..

[38] Carucci L., Nocerino R., Paparo L., De Filippis F., Coppola S., Giglio V., Cozzolino T., Valentino V., Sequino G., Bedogni G. (2022). Therapeutic effects elicited by the probiotic *Lacticaseibacillus rhamnosus* GG in children with atopic dermatitis. The results of the ProPAD trial. Pediatr. Allergy Immunol..

[39] Chan O.M., Xu W., Cheng N.S., Leung A.S.Y., Ching J.Y.L., Fong B.L.Y., Cheong P.K., Zhang L., Chan F.K.L., Ng S.C. (2024). A novel infant microbiome formula (SIM03) improved eczema severity and quality of life in preschool children. Sci. Rep..

[40] Cukrowska B., Ceregra A., Maciorkowska E., Surowska B., Zegadło-Mylik M.A., Konopka E., Trojanowska I., Zakrzewska M., Bierła J.B., Zakrzewski M. (2021). The Effectiveness of Probiotic *Lactobacillus rhamnosus* and *Lactobacillus casei* Strains in Children with Atopic Dermatitis and Cow’s Milk Protein Allergy: A Multicenter, Randomized, Double Blind, Placebo Controlled Study. Nutrients.

[41] Becheanu C.A., Ţincu I.F., Smădeanu R.E., Coman O.A., Coman L., Ţincu R.C., Păcurar D. (2019). Benefits of Oligofructose and Inulin in Management of Functional Diarrhoea in Children—Interventional Study. Farmacia.

[42] Rosali M.I., V Thanga Velu D.P., Mokhtar M.H., Raja Ali R.A., Mohd Mokhtar N., A Hamid A. (2026). Specificity vs. Synergy Between Single-Strain and Multi-Strain Probiotics for Ulcerative Colitis Treatment: A Review of the Literature. Biomedicines.

[43] Chan C.W.H., Chan J.Y.W., Leung T.F., Choi K.C., Tsui S.K.W., Wong C.L., Chow K.M. (2020). Altered Gut Microbiome and Environmental Factors Associated with Development of Eczema in Hong Kong Infants: A 4-Month Pilot Study. Int. J. Environ. Res. Public Health.

[44] Lee M.J., Kang M.J., Lee S.Y., Lee E., Kim K., Won S., Suh D.I., Kim K.W., Sheen Y.H., Ahn K. (2018). Perturbations of gut microbiome genes in infants with atopic dermatitis according to feeding type. J. Allergy Clin. Immunol..

[45] Blicharz L., Samborowska E., Zagożdżon R., Bukowska-Ośko I., Czuwara J., Zych M., Roszczyk A., Perlejewski K., Makowska K., Nowaczyk J. (2025). Severity of atopic dermatitis is associated with gut-derived metabolites and leaky gut-related biomarkers. Sci. Rep..

[46] Blicharz L., Samborowska E., Zagożdżon R., Czuwara J., Zych M., Roszczyk A., Zaremba M., Dadlez M., Samochocki Z., Olszewska M. (2025). Food Sensitization Is Associated with Atopic Dermatitis Severity, Gut-Derived Metabolites and Leaky Gut in Adults. Clin. Transl. Allergy.

[47] Endres L.M., Bungau A.F., Tit D.M., Bungau G.S., Radu A., Diaconu C.C., Marin R.C. (2025). Acne Vulgaris Associated with Metabolic Syndrome: A Three-Case Series Highlighting Pathophysiological Links and Therapeutic Challenges. Diagnostics.

[48] Stokholm J., Blaser M.J., Thorsen J., Rasmussen M.A., Waage J., Vinding R.K., Schoos A.M., Kunøe A., Fink N.R., Chawes B.L. (2018). Maturation of the gut microbiome and risk of asthma in childhood. Nat. Commun..

[49] Galazzo G., van Best N., Bervoets L., Dapaah I.O., Savelkoul P.H., Hornef M.W., GI-MDH Consortium, Lau S., Hamelmann E., Penders J. (2020). Development of the Microbiota and Associations with Birth Mode, Diet, and Atopic Disorders in a Longitudinal Analysis of Stool Samples, Collected from Infancy Through Early Childhood. Gastroenterology.

[50] Zimmermann P., Messina N., Mohn W.W., Finlay B.B., Curtis N. (2019). Association between the intestinal microbiota and allergic sensitization, eczema, and asthma: A systematic review. J. Allergy Clin. Immunol..

[51] Pantazi A.C., Nori W., Kassim M.A.K., Balasa A.L., Mihai C.M., Chisnoiu T., Mihai L., Ungureanu A., Frecus C.E., Chirila S.I. (2024). Gut microbiota profile and atopic dermatitis in the first year of life. J. Med. Life.

[52] Łoś-Rycharska E., Gołębiewski M., Sikora M., Grzybowski T., Gorzkiewicz M., Popielarz M., Gawryjołek J., Krogulska A. (2021). A Combined Analysis of Gut and Skin Microbiota in Infants with Food Allergy and Atopic Dermatitis: A Pilot Study. Nutrients.

[53] Czarnowicki T., He H., Krueger J.G., Guttman-Yassky E. (2019). Atopic dermatitis endotypes and implications for targeted therapeutics. J. Allergy Clin. Immunol..

