# Supplementary material for: Clinical Outcomes Following Personalized Gut Microbiota-Targeted Therapy in Children with Atopic Dermatitis and Laboratory-Confirmed Dysbiosis: A Single-Arm Prospective Pilot Study
Source: Nutrients. 2026 Aug 6;18(15):2572. doi: 10.3390/nu18152572 (PMC13468685; doi:10.3390/nu18152572)
Supplement: Supplementary file 1 [file nutrients-18-02572-s001.zip › Supplementary Note S1.pdf]

Supplementary Note S1. Supplementary Statistical Analyses Supporting the Main Findings

This Supplementary Note provides additional statistical analyses supporting the principal findings presented in the main manuscript. In accordance with reviewer recommendations, emphasis is placed on effect sizes, confidence intervals, responder analyses, and complementary robustness assessments rather than post-hoc (observed) statistical power. Unless otherwise indicated, all analyses are exploratory and should be interpreted in conjunction with the limitations discussed in the main text.

S1.1. Effect Sizes and Clinical Significance

Outcome	Baseline (mean ± SD)	90 days (mean ± SD)	Mean change (95% CI)	Relative reduction	Cohen's d_z	Partial η^2 (time)	MCID attained
POEM	14.67 ± 5.22	7.10 ± 3.82	−7.57 (5.45–9.69)	≈52%	2.03	0.759	18/21 (85.7%)
SCORAD	41.66 ± 15.28	17.93 ± 11.68	−23.73 (16.59–30.86)	≈57%	1.90	0.749	18/21 (85.7%)

*d*_z, paired Cohen's *d*; partial η^2, omnibus within-subject time effect from repeated-measures ANOVA; MCID, minimal clinically important difference (POEM ≥3.4 points; SCORAD ≥8.7 points). Both mean relative reductions exceeded the established MCID thresholds.

S1.2. Responder Analyses (90 days, N = 21)

Definition	POEM	SCORAD
≥50% reduction (primary)	16/21 (76.2%)	15/21 (71.4%)
≥30% reduction	19/21 (90.5%)	19/21 (90.5%)
MCID (POEM ≥3.4 / SCORAD ≥8.7 points)	18/21 (85.7%)	18/21 (85.7%)
Final score in mild/clear range (POEM ≤7 / SCORAD <20)	14/21 (66.7%)	13/21 (61.9%)
≥70% reduction (stringent)	3/21 (14.3%)	—

Responder rates did not differ significantly according to High Putrefaction Flora status (POEM 73% vs 83%, Fisher's exact p = 1.000; SCORAD 60% vs 100%, p = 0.123). These subgroup analyses involved very small sample sizes and should therefore be regarded as exploratory and inconclusive.

S1.3. Repeated-Measures ANOVA and Bonferroni-Adjusted Pairwise Comparisons

Omnibus time effect

- POEM: $F(2,40) = 63.04$, $p < 0.001$, partial $\eta^2 = 0.759$ (Mauchly $W = 0.740$, $p = 0.057$)
- SCORAD: $F(1.39,27.71) = 59.56$, $p < 0.001$, partial $\eta^2 = 0.749$ (Mauchly $W = 0.556$, $p = 0.004$; Greenhouse–Geisser $\varepsilon = 0.693$)

Contrast	POEM mean difference (95% CI)	*p*	SCORAD mean difference (95% CI)	*p*

Baseline − 30 days	4.90 (3.07–6.74)	<0.001	15.64 (9.61–21.68)	<0.001
Baseline − 90 days	7.57 (5.45–9.69)	<0.001	23.73 (16.59–30.86)	<0.001
30 days − 90 days	2.67 (1.36–3.97)	<0.001	8.08 (4.51–11.66)	<0.001

Pairwise comparisons were performed using paired *t*-tests with Bonferroni-adjusted 95% confidence intervals.

S1.4. Association of Dysbiosis Markers with Disease Severity and Clinical Improvement

Children with High Putrefaction Flora (HPF+, n = 15) had significantly higher POEM scores than HPF-negative participants (n = 6) at baseline (15.0 vs 9.0; p = 0.023), 30 days (12.0 vs 7.0; p = 0.018), and 90 days (7.0 vs 4.5; p = 0.045). SCORAD differed significantly only at Day 90 (20.7 vs 11.4; p = 0.037).

The continuous Flora Index showed no statistically significant association with baseline disease severity (POEM ρ = −0.11, p = 0.64; SCORAD ρ = −0.18, p = 0.45) or with clinical improvement (ΔPOEM ρ = 0.03, p = 0.89; ΔSCORAD ρ = −0.08, p = 0.72).

Between-group improvement also did not differ significantly (ΔPOEM 8.20 ± 4.14 vs 6.00 ± 1.79, p = 0.184, Cohen's d = 0.60; ΔSCORAD 23.32 ± 13.88 vs 24.75 ± 9.20, p = 0.938, Cohen's d = −0.11). These subgroup comparisons should be interpreted as exploratory and inconclusive because of the limited sample size.

S1.5. Complementary Sensitivity Analyses

Exclusion of the single clear non-responder (participant 3) resulted in only minimal changes in the mean improvement (ΔPOEM 7.57 → 7.80; ΔSCORAD 23.73 → 24.53), and all within-subject baseline-to-90-day comparisons remained statistically significant (p < 0.001).

Children with higher baseline disease severity (SCORAD >40; n = 10) experienced larger absolute improvements than those with lower baseline severity (n = 11) (mean ΔPOEM 10.0 vs 5.4; mean ΔSCORAD 32.1 vs 16.1).

These findings indicate that the principal results were not driven by a single influential observation.

S1.6. Complementary Evaluation of the Regression Models

Bootstrap resampling (5000 replications) of the Model 2 standardized Flora Index coefficient yielded confidence intervals spanning zero (POEM β = −0.03, 95% CI −0.34 to 0.34; SCORAD β = −0.06, 95% CI −0.35 to 0.39).

Ridge regression reproduced the dominance of baseline disease severity (POEM baseline 0.53, Flora −0.04; SCORAD baseline 0.30, Flora −0.08).

Leave-one-out cross-validated R^2 decreased as predictors were added (POEM: M1 0.37 → M2 0.29 → M3 0.14; SCORAD: M1 0.12 → M2 0.05 → M3 −0.08), indicating no consistent independent predictive contribution of the Flora Index and confirming the exploratory nature of Model 3.

Together, these complementary analyses support the conclusion that baseline disease severity remained the dominant predictor of short-term clinical outcomes, whereas the evaluated culture-based dysbiosis markers did not demonstrate consistent independent prognostic information in this exploratory cohort. Given the limited sample size, these findings should be interpreted as inconclusive rather than as evidence that the evaluated microbiological markers lack independent prognostic relevance.

S1.7. Illustrative Sample-Size Estimation for a Future Randomized Controlled Trial

Using the observed effect sizes, a two-arm parallel randomized controlled trial (80% power, α = 0.05, two-sided, 1:1 allocation) would require approximately 64, 45, and 26 participants per arm to detect standardized between-group differences of Cohen's d = 0.5, 0.6, and 0.8, respectively.

Incorporating baseline disease severity as a covariate (ANCOVA; observed baseline–outcome correlation $\rho \approx 0.6$) reduces these estimates to approximately 41 and 29 participants per arm for Cohen's $d = 0.5$ and 0.6, respectively.

Allowing for approximately 20% attrition, a target sample size of **55–80 children per arm (total approximately 110–160 participants)** is recommended for a definitive randomized controlled trial.
