# Supplementary material for: Clinical Outcomes Following Personalized Gut Microbiota-Targeted Therapy in Children with Atopic Dermatitis and Laboratory-Confirmed Dysbiosis: A Single-Arm Prospective Pilot Study
Source: Nutrients. 2026 Aug 6;18(15):2572. doi: 10.3390/nu18152572 (PMC13468685; doi:10.3390/nu18152572)
Supplement: Supplementary file 1 [file nutrients-18-02572-s001.zip › Supplementary Table S1.pdf]

Supplementary Table S1. Individualized microbiota-targeted therapy regimens and baseline microbiological findings (N = 21).

Pt	Sex/Age (y)	Depleted (acidifying) taxa at baseline	Proteolytic / abnormal findings	FI	Dysbiosis	Individualized regimen prescribed
1	M/2	Lactobacillus spp.; Enterococcus spp.	no	3	mild	Lactobacillus spp. , Enterococcus spp.
2	F/2	Enterococcus spp.	Escherichia coli spp.	3	mild	Enterococcus spp.
3	F/7	Bacteroides spp.; Enterococcus spp.;	Escherichia coli spp.	3	mild	Bacteroides spp. , Enterococcus spp.
4	F/6	Lactobacillus spp. Enterococcus spp.	Escherichia coli spp.	7	intermediate	Lactobacillus spp. , Enterococcus spp.
5	F/8	Enterococcus spp.	Escherichia coli spp.	4	mild	Enterococcus spp.
6	M/5	Enterococcus spp.	Candida albicans	5	mild	Enterococcus spp. fluconazol
7	F/3	Escherichia coli spp.; Bacteroides spp.; Enterococcus spp.	Candida albicans	6	intermediate	Bacteroides spp. ; Enterococcus spp.; fluconazole
8	M/2	Lactobacillus spp.; Enterococcus spp.	Escherichia coli spp.; Proteus spp.	7	intermediate	Lactobacillus spp., Enterococcus spp.
9	M/2	Enterococcus spp.	high PH	3	mild	Enterococcus spp.;
10	F/15	Lactobacillus spp.; Enterococcus spp.	Escherichia coli spp.; high PH	6	intermediate	Lactobacillus spp. , Enterococcus spp.
11	F/5	Enterococcus spp.	Escherichia coli species; Clostridium	5	mild	Enterococcus spp.
12	F/13	Enterococcus spp.	clostridium species	4	mild	Enterococcus spp.
13	M/8	Lactobacillus spp.; Enterococcus spp.; Bacteroides spp.	Escherichia coli species;	6	intermediate	Lactobacillus spp. , Enterococcus spp., Bacteroides spp.

14	M/1	Lactobacillus spp.; Enterococcus spp.	Escherichia coli spp.; high PH	6	intermed iate	Lactobacillus spp. , Enterococcus spp.; Bacteroides spp.
15	M/5	Lactobacillus spp., Bifidobacterium spp.s	Escherichia coli species; high PH	5	mild	Lactobacillus spp., Bifidobacterium spp.
16	M/2	Lactobacillus strains	Clostridium spp.	2	mild	Lactobacillus spp., Bifidobacterium spp.
17	F/1	Lactobacilli spp.; Enterococci spp.	E. Coli, Klebsiella, Enterobacter spp.	9	intermed iate	inulin, Bifidobacterium spp.; Streptococcus thermophilus, Lactobacillus spp., Enterococi spp.,
18	M/3	Bifidobacterium spp., Lactobacillus spp., Enterococcus spp.	—	4	mild	Bifidobacterium, spp. Lactococcus spp., Lactobacillus spp., Enterococcus spp.
19	F/3	Bifidobacterium spp., Lactobacillus spp., Enterococcus spp.	Candida albicans	6	intermed iate	Bifidobacterium spp., Lactobacillus spp., Enterococcccus spp., inuline
20	F/3	Bacteroides spp.; Enterococcus spp.; Lactobacillus spp.	Escherichia coli spp., Clostridum, Candida albicans	7	intermed iate	Bifidobacterium spp., Lactobacillus spp. , Enterococcccus spp.; fluconazole
21	M/6	Enterobacter spp.; Bifidobacter spp.	high PH	5	mild	Bifidobacterium spp., Lactobacillus spp. , Enterococcccus spp.

FI, Flora Index; laboratory-defined proprietary composite ordinal score, with higher values indicating greater microbiological dysbiosis. Dysbiosis categories (mild/intermediate) and FI values were assigned by the reference laboratory (MVZ GANZIMMUN GmbH, Mainz, Germany) using a proprietary algorithm that is not publicly available. Regimens were individualized according to each participant's stool microbiological profile and delivered alongside routine dermatological care. Probiotic genera are reported at the genus level because exact strains, commercial formulations, colony-forming-unit doses, and treatment durations were not standardized or systematically recorded. Oral fluconazole was prescribed only for participants with documented fungal overgrowth, whereas inulin denotes prebiotic supplementation.
