# Supplementary material for: Clinical Outcomes Following Personalized Gut Microbiota-Targeted Therapy in Children with Atopic Dermatitis and Laboratory-Confirmed Dysbiosis: A Single-Arm Prospective Pilot Study
Source: Nutrients. 2026 Aug 6;18(15):2572. doi: 10.3390/nu18152572 (PMC13468685; doi:10.3390/nu18152572)
Supplement: Supplementary file 1 [file nutrients-18-02572-s001.zip › Supplementary Table S2.pdf]

Supplementary Table S2. Complete individual participant-level data (N = 21).

ID	Sex	Age (y)	C/S	Preterm	IVF	BF	HPF	FI	Dysbiosis	POEM 0	POEM 30	POEM 90	SCORAD 0	SCORAD 30	SCORAD 90
1	M	2	Yes	Yes	No	No	No	3	mild	17	7	8	52.25	23	10.8
2	F	2	Yes	Yes	Yes	No	Yes	3	mild	15	9	5	53.1	28.55	15.55
3	F	7	Yes	No	No	No	Yes	3	mild	21	15	18	67.2	57.45	59.45
4	F	6	Yes	No	No	Yes	Yes	7	intermediate	24	16	11	70.7	29.6	22.6
5	F	8	Yes	No	No	No	Yes	4	mild	18	7	5	52.8	26.9	21.6
6	M	5	Yes	No	No	Yes	No	5	mild	8	4	3	30.2	14	11.95
7	F	3	Yes	No	No	Yes	No	6	intermediate	9	3	2	35.2	10.2	8.45
8	M	2	Yes	No	No	No	Yes	7	intermediate	10	3	2	37.35	11.4	8.05
9	M	2	Yes	Yes	No	No	No	3	mild	9	7	4	27.1	19.2	8.9
10	F	15	No	No	No	No	Yes	6	intermediate	19	12	7	55.75	42.25	20.7
11	F	5	Yes	Yes	No	No	Yes	5	mild	16	12	8	44.45	29.95	17.7

12	F	13	No	No	No	Yes	Yes	4	mild	24	18	12	63.3	37.95	25.45
13	M	8	No	No	No	Yes	Yes	6	intermediate	12	8	6	24.45	17.45	1.95
14	M	1	Yes	Yes	No	No	Yes	6	intermediate	22	12	8	51	34	22
15	M	5	Yes	Yes	No	Yes	No	5	mild	12	10	6	40.75	32.9	14.4
16	M	2	Yes	No	No	Yes	No	2	mild	9	7	5	31.05	17.05	13.55
17	F	1	Yes	No	No	No	Yes	9	intermediate	7	6	6	13.95	9.45	8.45
18	M	3	Yes	Yes	No	Yes	Yes	4	mild	14	10	7	32.2	20.7	17.2
19	F	3	Yes	No	No	Yes	Yes	6	intermediate	15	16	13	30.85	32.75	29.25
20	F	3	Yes	Yes	No	No	Yes	7	intermediate	13	11	7	33.2	26.2	22.4
21	M	6	Yes	Yes	No	No	Yes	5	mild	14	12	6	28	25.4	16.2

C/S, cesarean section; BF, any breastfeeding; HPF, High Putrefaction Flora; FI, Flora Index. POEM 0/30/90 and SCORAD 0/30/90 denote scores at baseline, 30 days, and 90 days. All 21 participants had complete data at every time point (complete-case; no missing values).
